# A convenient workflow to spot photosensitizers revealed photo-activity in basidiomycetes†‡

**DOI:** 10.1039/c8ra10181g

**Published:** 2019-02-05

**Authors:** Bianka Siewert, Pamela Vrabl, Fabian Hammerle, Isabella Bingger, Hermann Stuppner

**Affiliations:** Institute of Pharmacy/Pharmacognosy, Center for Molecular Biosciences Innsbruck (CMBI), Center for Chemistry and Biomedicine, University of Innsbruck Innrain 80-82 Innsbruck 6020 Austria Bianka.siewert@uibk.ac.at; Institute of Microbiology, University of Innsbruck Technikerstraße 25d Innsbruck 6020 Austria; Management Center Innsbruck Maximilianstraße 2 Innsbruck 6020 Austria

## Abstract

Photodynamic therapy (PDT) is an alternative approach for the treatment of neoplastic diseases employing photosensitizers activated by light. In order to discover new natural photosensitizers, a convenient workflow was established. To validate the workflow, fungi were selected, because we hypothesized that fruiting bodies and mycelia are an overlooked source. The results proved the hypothesis, as exorbitant high photo-cytotoxicity values were detected. For example, the acetone extract of *Cortinarius croceus* was characterized by an EC_50, 9.3 J cm^−2^_ of 1 µg mL^−1^ against cells of a lung cancer cell-line (A549). In sum, a low-cost workflow for the detection and biological evaluation of photosensitizers is presented and discussed. Furthermore, this paper provides the first experimental evidence for phototoxic metabolites in basidiomycetes. This hints towards a new assignable function of fungal pigments, *i.e.* photochemical defense.

## Abstract 2

An expedient approach to spot natural photosensitizers was established and with it were – for the first time – photosensitizers in mushrooms identified.

## Graphical abstract

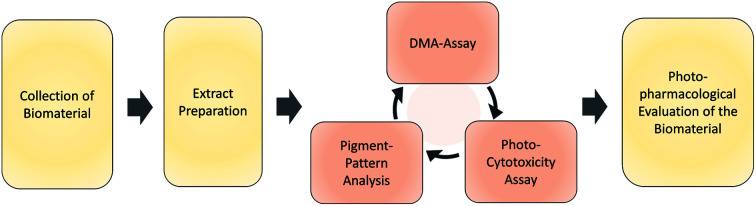

## Introduction

In India's sacred book Atharva-Veda, dated back to around 3500 BC, the sun-worshipping prayer is recorded as a fundamental part of medicinal treatments.^1^ Today we know this prayer leads to the inevitable sun exposure needed to activate *e.g.* the ingredients of *Psoralea corylifolia*.^1^ Such light-combined therapies are, however, not restricted to India's ethnopharmaceutical knowledge. They are also found in many other ancient civilizations^2^ and are clinically implemented since the early nineties.^3^

The major benefit of the so-called photodynamic therapy (PDT) is its selectivity: only illuminated cells are affected, cells or tissues left in the dark remain unharmed. This selectivity permits spatial controlled therapies and allows a higher therapeutic window while minimizing the threatening adverse-effects of classic therapies.^4^ Nevertheless, despite promising clinical results,^5^ PDT and antimicrobial photodynamic inhibition (aPDI)^6^ are not widely applied clinical therapies.^7^ Hampering factors are based on the light sources and the limited amount of approved photosensitizers. The revolution in lighting techniques, however, let limitations based on irradiation issues disappear.^8,9^ Optical fibers are nowadays used to transmit light. Such fibers can be implanted directly into the tumour permitting *e.g.* interstitial illuminations.^5^ Thus, tissue-penetration-depth issues based on the irradiation wavelength vanished and therefore the optical window of PDT broadened. Still missing, however, are new chemical entities. Therefore, this work focuses on establishing a tool to detect new lead-compounds for PDT and aPDI.

Nature might be the origin of new PS. Known classes of natural PS are of herbal origin and include perylenequinones with hypericin from St John's wort (*Hypericum perforatum*), furanocoumarins with psoralen from fool's parsley (*Aethusa cynapium*),^10^ phenalenones with lachnanthocarpone from paint-root (*Lachnanthes tinctoria*),^11,12^ and β-carboline alkaloids with harmane from the Chinese plant *Evodia rutaecarpa*.^12^ Moreover, anthraquinones from Argentinian shrubs (*Heterophylla* spp.) were identified as natural PSs, *e.g.* soranjidiol.^13^ From a chemical point of view, several fungal metabolites appear to be analogue structures or are precursors of known herbal PS. Austroventin, for example, is oxidized to hypericin when insects attack the fruiting bodies of *Cortinarius austrovenetus* (=*Dermocybe austroveneta*).^14^ Other webcap (*Cortinarius*) species are known to produce a vast number of anthraquinones, *e.g.* questin, emodin, or 7,7′-emodinphyscion.^15^ Anthraquinones belong to the top ten photosensitizers,^16^ but also alkaloids analogue to the PS harmane were isolated from fungi, *e.g.* infractin from *C. infractus.*^17^ Funalenone, a phenalenone similar to the one in paint-roots,^18^ is produced by several *Aspergillus* species like *A. tubingensis*^19^ or *A. niger*.^11^ Considering the structural similarities, we thus hypothesize that the kingdom fungi is an underestimated source for PS and decided to employ fungi as sample source to validate our workflow. Exemplary with selected fungi, we introduce the principles of our workflow, discuss the rationale behind the implemented assays, and provide an interpretation-guideline for the assessment of the source's photo-therapeutic potential.

## Results and discussion

**Fig. 1:**
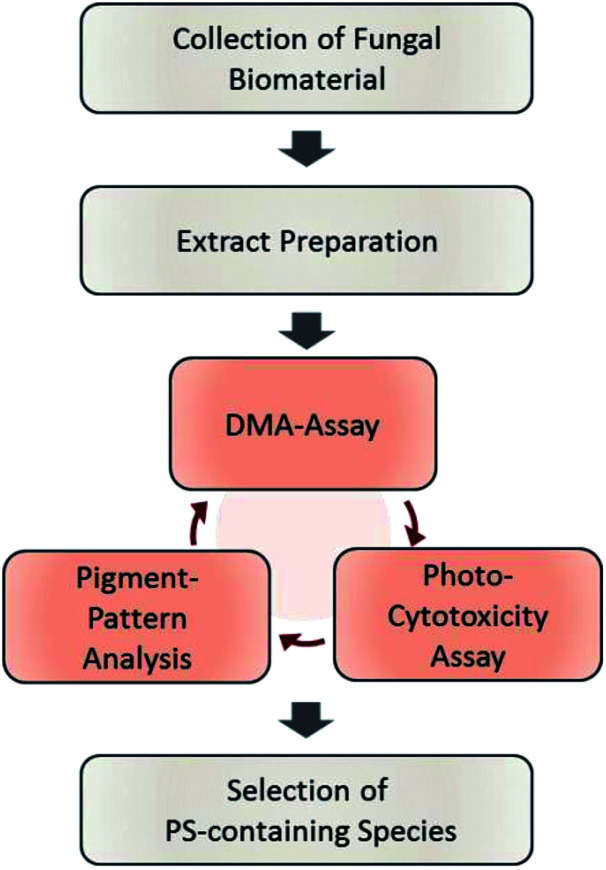
Flowchart of the steps involved in the established phototoxicity screening workflow. After sampling an adequate amount of the natural source, extracts of each are prepared. The prepared extracts are submitted to the DMA-assay, the photo-cytotoxicity-assay, and the pigment-pattern analysis. The order of these investigations is not fixed; the conclusion will be drawn from the combined results of all three sub-assays. For an interpretation guideline, see Fig. 4.

The designed workflow (Fig. 1) consists out of three assays. A chemical assay based on dimethyl-anthracene (DMA) indicates whether singlet oxygen (^1^O_2_) is produced due to the irradiation of an extract or not. *Via* an analytical assay based on the HPLC-DAD-MS technology insights are gained in the chemical profile of possible pigments responsible for the photo-effect. Finally, an *in vitro* photo-cytotoxicity assay is conducted to rank the biological relevance.

### The DMA-assay

Clinically most relevant are PDT Type II sensitizers producing ^1^O_2_ while being irradiated. For the detection of ^1^O_2_ several chemical probes are known.^20,21^ Out of them, DMA was chosen as inexpensive and selective agent.^22^ The selectivity is based on its detection mechanism, which is a photosensitized oxidation of the anthracene-core leading to a disruption of the conjugated π-system (see Fig. S2‡).

This in turn, suppresses the typical absorption of DMA (*λ*_max, EtOH_ = 377 nm) as well as emission. The extent of quenching, measured by the ΔOD_377 nm_ can be correlated to the relative ^1^O_2_ production by using a reference PS *via* equation (eqn (1)) (detailed explanation in the ESI‡).1
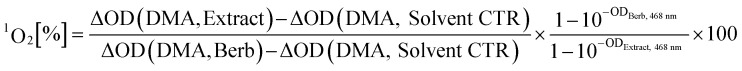


The reference PS should be aligned to the colour of the investigated extracts and the available light source. For red-light absorbing blue pigments, one would use *e.g.* methylene blue (*λ*_irr_ = 632 nm, *ϕ*_1O2, EtOH_ = 0.49)^23^ or hypericin (*λ*_irr_ = 590 nm, *ϕ*_1O2, PB_ = 0.49)^23^ as reference for the production of ^1^O_2._ For green-light absorbing red pigments, Rose bengal (*λ*_irr_ = 555 nm, *ϕ*_1O2, EtOH_ = 0.86)^23^ would be a good choice. In this study, the blue light absorbing natural photosensitizer berberine (*λ*_max_ = 420 nm, *ϕ*_1O2, DCM_ = 0.25, yellow pigment)^23^ was chosen in accordance to the colour of the fungal extracts (yellow to orange) and the blue irradiation panel (468 ± 27.3 nm). A PS of natural origin was preferred over a synthetic one, as in this way a plant extract containing the reference PS could be included allowing to rank the obtained results. Berberine, however, can not only produce ^1^O_2_ (Type II) it may also act itself as radical (Type I PDT).^24^ In the setting of the DMA assay, solely the ^1^O_2_ production capacity of berberine was utilized, which is guaranteed by the selective probe. The DMA-assay itself was conducted in 96 well-plates allowing a medium-size throughput. For each extract, the measurement of several experiments before and after irradiation was necessary. First, the DMA-absorbance (*λ*_abs_ = 377 nm) was measured to detect singlet oxygen. Second, the optical density of the pure extract at the irradiation wavelength (*λ*_abs_ = 468 nm) was recorded to correct the singlet oxygen production by the probability of absorption. Third, a co-incubation with a ^1^O_2_ quencher (*i.e.* ascorbic acid) was done to exclude false positive results. As the experiment is of chemical nature, and not of a more complex biological,^21^ one can conclude that ^1^O_2_ was produced if the consumption of DMA is quenched by adding a scavenger. Finally, a control experiment containing solely the extract and ascorbic acid was included to spot any unwanted side-reaction.

**Fig. 2:**
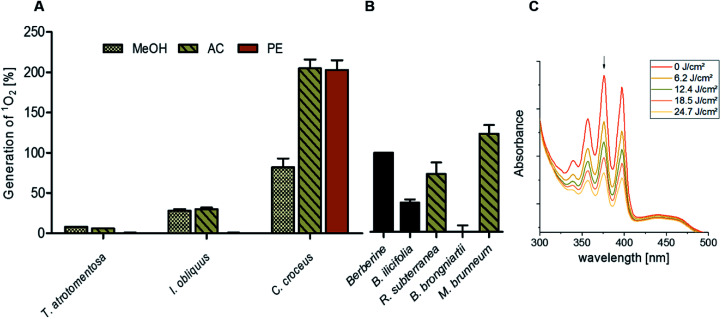
Relative singlet oxygen production of selected fungal extracts calculated by illuminating the samples in ethanol with a light dose of 24.7 J cm^−2^ (468 nm, 20.6 mW cm^−2^). (A) The basidiomycetes *T. atrotomentosa*, *I. obliquus*, and *C. croceus* as well as (B) the ascomycetes *R. subterranean*, *B. brongniartii*, and *M. brunneum*. The PS berberine was set to 100% as positive control, the extract of *B. ilicifolia* roots containing berberine yielded 37 ± 4%. The relative yields are given with standard error. (C) Evolution of the DMA absorption quenching due to the blue light irradiation (468 nm, 20.6 mW cm^−2^, light doses as indicated in the legend) exemplified with the acetone extract of *C. croceus*.

By submitting the extracts of the selected fungi to the DMA-assay intriguing insights were obtained: most of the basidiomycete extracts were able to produce ^1^O_2_ (Fig. 2). Especially all extracts of *C. croceus* seemed to contain PSs as shown by their significant ability to produce ^1^O_2_: the AC and the PE fractions, resulted in 205 ± 11% and 205 ± 12% ^1^O_2_ formation, respectively, and were thus more efficient than pure berberine (set to 100%). The methanol extract yielded 82 ± 11% singlet oxygen, which is still higher than the plant extract containing the known PS berberine (*i.e.* the root extract of *B. ilicifolia* yielding 37 ± 4% singlet oxygen). An activity similar to the *Berberis* extract was shown by the MeOH and AC extract of *I. obliquus* (*i.e.* 28 ± 2% and 30 ± 2%). The PE extract of *T. atrotomentosa* showed no activity, but the MeOH and AC fraction at least a minimal one (*i.e.* 6% each). Two of the three investigated ascomycetes showed a higher activity than the plant extract. In detail, *R. subterranea* produced 73 ± 15% singlet oxygen and for *M. brunneum* an activity of 124 ± 11% was found. In contrast, the extract of *B. brongniartii* showed no ^1^O_2_ production.

### HPLC pigment profiling

A HPLC-DAD-MS analysis was implemented in the workflow to judge whether the extract is a promising sources to isolate natural PSs. Therefore, the extract was monitored with the standard wavelength 210, 254, and 280 nm as well as by the wavelength of light irradiation. The first set of wavelengths is intended to judge the complexity of the chemical matrix and thereby the ease of isolation. The latter is necessary to gain information about the number of pigments being potentially responsible for the singlet oxygen production.

In this study, the fungal extracts were monitored at a wave light of 468 nm according to the emission maximum of the available irradiation setup. For a chemical profiling of the matrix and the pigments, the full electron absorption spectrum (210–800 nm) of each peak was additionally recorded. For all basidiomycetes, the majority of the pigments was found in the acetone fraction (Fig. S3‡). Based on the obtained mass, the UV-Vis spectra, and literature data, the main pigments of *T. atrotomentosa* and *C. croceus* could be identified as atromentin and 7,7′ biphyscion (refer to the ESI‡ for a detailed discussion).

For the ascomycetes, a suitable separation was obtained with a stationary phase containing phenyl-hexyl ligands leading to a selectivity for polar and aromatic compounds. The obtained spectra, however, revealed a systematic problem existing for samples with limited biomaterial, as *e.g.* those derived from Petri dishes: the concentration of the interesting metabolites was comparatively low, resulting in an intensity of the peaks close to the subjective LOQ. This effect is a direct consequence of the limited net mass of the biomaterial. With this limitation in mind, an interpretation of the HPLC-MS experiment was done and oosporein assigned for *B. brongniartii*. For a complete discussion, please refer to the ESI.‡

### (Photo)-cytotoxicity

In order to validate the results of the DMA-assay *in vitro*, all investigated extracts were tested regarding their photo-cytotoxic activity against the lung cancer cell line A549 and the cervical carcinoma cell line HeLa. 24 hours after drug treatment, the cells were irradiated in drug-free medium. As endpoint-assay the SRB-assay^25^ was preferred over the common used MTT,^26^ as MTT (and analogues) led to false-positive results (data not shown) due to their general redox-activity.^27,28^

**Fig. 3:**
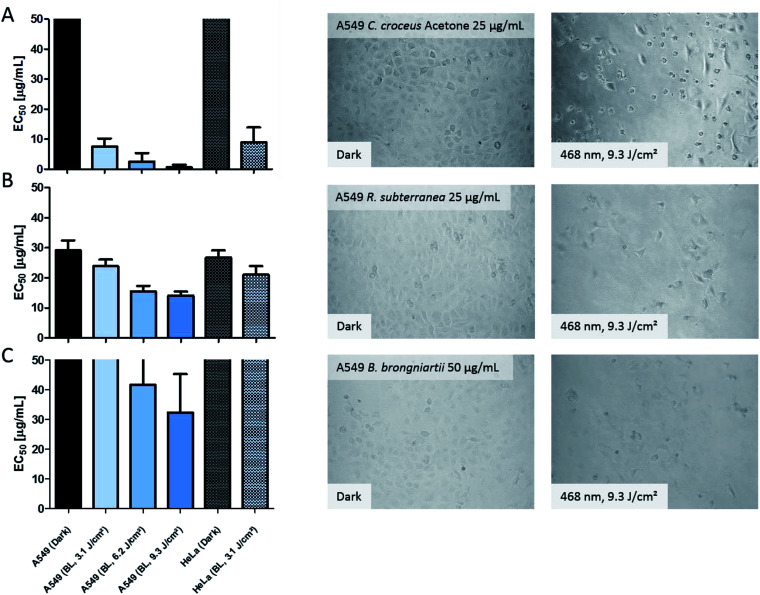
Photo-cytotoxicity results of the most promising extracts against A549 and HeLa cancer cells in the dark and under blue light irradiation. Bars: EC_50_ values with confidence interval (95%) if a reduction of the cell population was induced by the treatment. Top cutted bars indicate that the extracts were not active below 50 µg mL^−1^. Pictures: evaluation of the morphological changes induced by the extracts. The utilized concentration is indicated in the dark picture and was the same for the irradiated experiment. Line (A) acetone extract of *C. croceus*, line (B) *R. subterranea*, and line (C) *B. brongniartii.* The complete set of the microscopic investigation is depicted in the ESI.‡

The obtained results of the fungal extracts are displayed in detail in Table S2.‡ All photo-cytotoxic active basidiomycete extracts showed an activity in the DMA-assay. However, not all of the DMA-active extracts were active in the biological context, implying that the cellular membrane functioned as effective barrier for some PS. In detail, the extracts of *T. atrotomentosa* showed no phototoxic effect at all. Just a weak cytotoxic effect (approx. 40 µg mL^−1^) independent from light irradiation was observed for the PE extract. The extracts of *I. obliquus* were characterized by the absence of any significant light toxicity as well. The results for the *Cortinarius* species (Fig. 3a) were the most promising; a selectivity factor (see Fig. S9‡) of over 50 was obtained for the acetone fraction, which was non-toxic in the dark.

The PE fraction showed also an impressive phototoxicity, it was, however, additionally cytotoxic in the dark. This might be due to membrane modulating fatty acids in the PE fraction leading to leaks in the cell membrane and therewith to a toxic effect detracting from the photo-activated effect of this fraction. Most likely, the high photo-cytotoxicity is based on 7,7′ biphyscion, an anthraquinone dimer, which was identified by HPLC-DAD-MS as main metabolite in the acetone and PE extracts.

The three ascomycete extracts were characterized by lower EC_50_ values (*i.e.* less activity) as compared to the basidiomycetes. This observation might be an artificial effect due to the nature of the acetone-only extract. Nevertheless, the extract of *R. subterranea* was characterized by a moderate activity in the dark (EC_50, Dark_ = 29 µg mL^−1^) and a photo-enhanced effect due to the irradiation with blue light. Interesting observations were made for *M. brunneum* and *B. brongniartii*: while the *M. brunneum*-extract was active in the DMA assay, no activity was observed in the phototoxicity assay. For *B. brongniartii*, instead, the opposite was. The absence of any activity for *M. brunneum* indicated that the metabolites do not permeate the membrane or that the respective PSs are not stable in the medium. For the photo-cytotoxic *B. brongniartii* extract (Fig. 3C), singlet oxygen (Fig. 2) can be excluded as key player. Thus, one of the other PDT mechanisms^29^ was induced. Irradiation experiments with pure oosporein (*B. brongniartii's* main pigment) showed that a photochemical reaction was induced (data not shown), which might be the reason behind the observed photo-cytotoxicity.

### Interpretation guide of the phototoxicity screening workflow

**Fig. 4:**
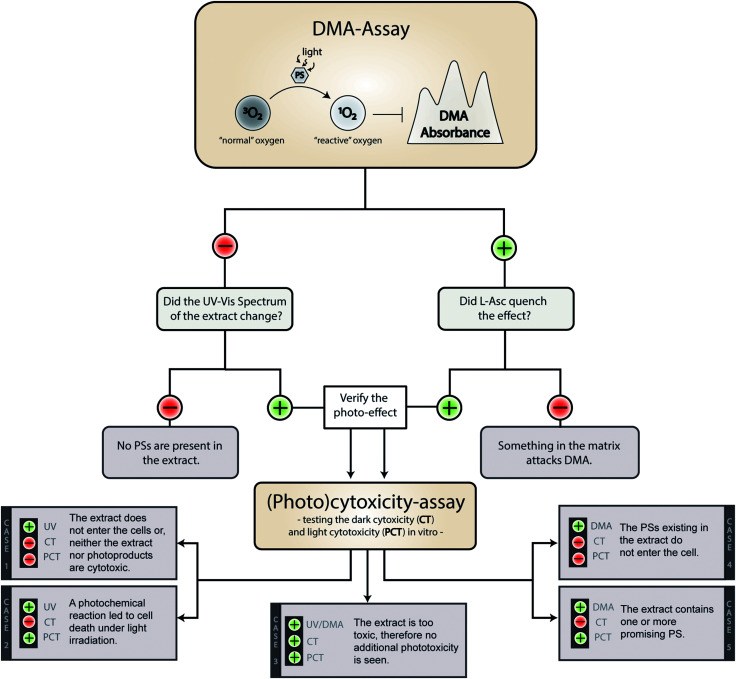
Possible outcomes of the established phototoxicity assay. With a green plus a positive result is marked, with a red minus a negative one. The following abbreviations were used: DMA (9,10-dimethyl anthracene), PS (photosensitizer), l-Asc (l-ascorbic acid), CT (cytotoxicity), PCT (photo-cytotoxicity), UV (evolution of the UV-Vis spectra).

On a meta-level, the presented phototoxicity workflow leads to a multi-layered result (see Fig. 4) which can be used to rank extracts from different natural sources (*e.g.* plants, algae, or fungi) regarding their phototoxic potential. An extract active in the DMA-assay is ranked by a photo-cytotoxicity assay with three potential outcomes: (a) no activity in the dark (CT ⊖), but in the light (PCT ⊕), (b) active in the dark (CT ⊕) and in the light (PCT⊕), or (c) inactive in the dark (CT ⊖) as well as under irradiation (PCT ⊖). Combining all information, following conclusions can be drawn in order to rank the extracts potential.

Case 1. The extract did not produce any ^1^O_2_, but showed a change in the UV-Vis spectra over the time of irradiation. Here the absence of any toxicity indicates a putative sun protecting action of the extract or simply the absence of any photobiologically relevant activity.

Case 2. The extract did not produce any ^1^O_2_, but was cytotoxic while being irradiated. In this case, one of the processes based on a photochemical mechanism was induced and the extract-matrix may contain a promising metabolite for the treatment of hypoxic tumours. In this study, the extract of *B. brongniartii* represents such a case and may therefore be an interesting candidate to develop oxygen independent PSs.

Case 3. The extract showed an activity on each level. This indicates that the extract might be too active and may lead to a systemic toxicity. Here it is recommended to carefully investigate the complexness of metabolites in the extract; an additional and/or synergistic effect of the ingredients might be present. The petrol ether extract of *C. croceus* fulfils these criteria, and as discussed above, matrix effects could be responsible. Thus, a detailed investigation of the extract should be done.

Case 4. The extract produced ^1^O_2_, but showed not photo-cytotoxicity. This might be a promising candidate for selective aPDI, in case it crosses bacterial or fungal membranes. The investigated extracts of *M. brunneum* showed this behaviour and should therefore be tested in more detail regarding their aPDI potential.

Case 5. The extract produced ^1^O_2_ and showed cytotoxicity solely under irradiation. Here a promising candidate for PDT-applications in mammalian might be present in the extract. In this preliminary study, the methanol extract of *C. croceus* achieved this promising behaviour. Therefore, it is subject of ongoing investigations.

Outlook. While this workflow selects fungi/plants containing unknown PDT-active metabolites, future research steps towards PDT-lead compounds are necessary. This steps should consider the following points: (1) the isolation and identification of the PS can be done *via* activity-guided isolation employing the DMA-assay. (2) The biological evaluation of the isolated metabolites should not only focus on the photo-cytotoxicity but also on the dark cytotoxicity. For the latter it is important to test the dark-effect against non-malignant cells in addition to the cancer cell line. These additional tests are important to gain preliminary information about potential side effects. (3) It might be that the isolated compound lacks its activity. In such a case, synergistic effects in the extracts were most likely responsible for the high ranking.

## Conclusions

In general, for the first time a low-cost, medium-throughput workflow to screen natural extracts for PS was presented. The workflow is not limited to fungal extracts. Plant extracts or pure compounds can be evaluated as well. As in general light-enhanced pharmaceutical actions of natural products tend to be neglected, a systematic exploration holds an impressive potential. Furthermore, the bioactivity-guided isolation of the photosensitizers is possible in standard phytochemical laboratories employing one part of the workflow, *i.e.* the DMA-assay. This in turn is a substantial requirement for an efficient isolation of new PS, which are mandatory for alternative antitumor or antimicrobial strategies in order to overcome existing resistances.

In detail, of the different fungal species exemplified, four appeared to produce PDT-type II PSs, while at least one seemed to contain another PS type. Neither one of the fungi was described as producer of PS before nor was one of the fungal pigments described as photoactive. For basidiomycetes, it is the first experimental evidence of photoactive metabolites. The existence of PS is of ecological interest as light-induced toxic effects belong to a yet unknown photochemical defence mechanism for these organisms. If such findings accumulate in the future, the discovery of photo activity will shed a new light on the physiological role of several fungal pigments, which otherwise – according to Spiteller^30^ – stays obscure.

## Material and methods

### Organisms and samples species

Petri dish cultures of *B. brongniartii* (BIPESCO 2) were grown on Sabouraud-2-Glucose agar medium at 25 °C for 9 days as described in the literature.^31^ Petri dish cultures of *M. brunneum* (BIPESCO 5) were cultivated on Sabouraud-4-Glucose agar medium at 25 °C for 12 days. *R. subterranean* were grown for approximately 4 weeks on PDA agar plates and kept at 4 °C until further use. Fruiting bodies of *C. croceus* and *T. atrotomentosa* were obtained in dry form from the herbarium in Innsbruck (kindly donated by U. Peintner and M. Kirchmair, University of Innsbruck, Austria) in 2017. Dried fruiting bodies of the orange black *I. obliquus* were kindly donated by P. L.'Archeveque (Forestia Inc, Canada) in 2017, and a voucher specimen (Herbarium nr: Chaga 2017) is stored in the herbarium of the Institute of Pharmacy/Pharmacognosy of the University of Innsbruck.

### Extraction of fruiting bodies (basidiomycetes)

The dried material of the basidiomycetes was milled to obtain finely granulated material for soxhlet extraction. In a first step, the fruiting bodies (exact amount see Table S1‡) were defatted in the dark with petrol ether (PE, 400 mL) for 6 h. Thereafter acetone (AC, 400 mL) and methanol (MeOH, 400 mL) were used under identical conditions. The yielded extracts were reduced under vacuum and stored in the dark. The amount of each obtained extract, the relative yield, and the colour are given in Table S1.‡ For all tests, an aliquot of the extract was solved in DMSO (final concentration of 10 mg mL^−1^) and filtrated through cotton wool.

### Extraction of Petri dish cultures (ascomycetes)

At least three individual colonies with the agar were cut in pieces, transferred into a centrifugation tube (50 mL) and subjected to an ultrasonic extraction (30 min) with acidified acetone (*ca.* 10 mL, 1 L acetone + 1 mL 2 N HCl). This different extraction method was chosen due to the limited biomaterial as compared to the fruiting bodies. After centrifugation (3000 rcf, 5 min), the supernatant was decanted and evaporated under a stream of air. The obtained yields and coloration of the extracts are listed in Table S1.‡ For analytical investigations, the extracts were solved in DMSO (final concentration 10 mg mL^−1^) and filtered through a membrane filter (Carl Roth, 0.45 µm, PTFE) before being subjected to any analysis.

### 96 LED-setup for illumination

An irradiation setup was built by Leiden University with marginal modifications to the published setup by Hopkins *et al.*^32^ For the present setup, LEDs with an emission of 468 nm (±27.3 nm) were utilized. A power density of 20.6 ± 1.1 mW cm^−2^ was calculated. A chemical actinometer, *i.e.* ferrioxalate, was utilized^32^ to confirm the homogenous irradiation across the inner 60 wells (Fig. S1‡).

### Chemical analysis of singlet oxygen (^1^O_2_) production

An ethanolic DMA solution was prepared (0.35 mM) and transferred (190 µL per well) into of a 96-well plate. A DMSO solution (10 µL, 1 mg mL^−1^) of each extract was added to the respective wells. The absorption of the extract alone (50 µM, ethanol) was measured as well as the suppression of the DMA absorbance in the presence of l-ascorbic acid (5 mM, pH 7.0–7.4). An additional control experiment (extract and l-ascorbic acid) was performed. DMSO (5% in ethanol) was used as solvent control. Berberine (50 µg mL^−1^, 149 µM) was used as positive control. Excitation was performed with the 96 LED setup, whereby four irradiation steps (468 nm, 5 min each, 6.2 J cm^−2^ each) were chosen. The measurement of the absorption was performed with a microplate reader (Tecan, Spark M10) and done in triplicate. The singlet oxygen production after 20 min (24.8 J cm^−2^) was calculated relative to berberine using formula (eqn (2)). A detailed discussion of this formula can be found in the ESI.‡

### (Photo)-cytotoxicity-assay and cell culture maintenance

Cells of the non-small lung cancer cell line A549 (ATCC, Sigma-Aldrich) and the cervical cancer cell line HeLa (kindly donated by A. Trockenbacher (Management Center Innsbruck, Austria)) were maintained in T-flasks (25 cm^2^) and MEM-medium containing FCS (10%) and penicillin/streptomycin (1%). The (photo)-cytotoxicity was done as published before^32^ and is explained in more detail in the ESI.‡ The selectivity indices (S.I.) express the ratio of cells killed under irradiation and cells killed in the dark. It is calculated from the EC_50_ values as expressed in formula (eqn (2)). For each investigated concentration a biological triplicate of technical triplicates were done.

2 S.I. = EC_50,Dark_(EC_50,Irradiated_)^−1^

## Author contributions

BS conceived the study and designed it with PV. PV cultured the ascomycete *B. brongniartii* and *M. brunneum*. BS extracted all fungal samples, performed together with FH and IB the assays, interpreted the results and designed all graphs with input from PV. BS and PV wrote the first draft of the manuscript. HS contributed to the material expenses and to the critical writing process. All authors contributed to the manuscript revision, read, and approved the submitted version.

## Conflicts of interest

There are no conflicts to declare.

## Abbreviation

AC: Acetone
CT: Cytotoxicity
DAD: Diode-Array-Detector
DMA: 9,10 Dimethylanthracene
EC: Effective concentration
FCS: Fetal calf serum
MeOH: Methanol
MTT: 3-(4,5-Dimethylthiazol-2-yl)-2,5-diphenyltetrazoliumbromid
PCT: Photo-cytotoxicity
PDT: Photodynamic therapy
aPDI: Antimicrobial photodynamic inhibition
PE: Petrolether
PS: Photosensitizer
SRB: Sulforhodamine B
SI: Selectivity index

## Supplementary Material

RA-009-C8RA10181G-s001
