# Supplementary material for: A convenient workflow to spot photosensitizers revealed photo-activity in basidiomycetes
Source: RSC Adv. 2019 Feb 5;9(8):4545–52. doi: 10.1039/c8ra10181g (PMC6394893; doi:10.1039/c8ra10181g)
Supplement: RA-009-C8RA10181G-s001 [file RA-009-C8RA10181G-s001.pdf]

Electronic Supplementary Material (ESI) for RSC Advances.
This journal is © The Royal Society of Chemistry 2019

Supporting Information

A Convenient Workflow to Spot Photosensitizers Revealed Photo-activity in Basidiomycetes

Bianka Siewert[1*], Pamela Vrabl[2], Fabian Hammerle[1], Isabella Bingger[1,3], and Hermann Stuppner[1]

[1]Institute of Pharmacy/Pharmacognosy, Center for Molecular Biosciences Innsbruck (CMBI), Center for Chemistry and Biomedicine, University of Innsbruck, Innrain 80-82, Innsbruck, 6020 Austria

[2]Institute of Microbiology, University of Innsbruck, Technikerstraße 25d, Innsbruck, 6020 Austria

[3]Management Center Innsbruck, Maximilianstraße 2, Innsbruck, 6020 Austria

Contents

1. General Experimental Procedures. 3
2. LED Irradiation Setup 3
3. Extraction 4
4. DMA-Assay 4
   4.1. Calculation of the Relative Singlet Oxygen Yield 5
5. HPLC-DAD Chromatograms 6
   5.1. Analytical Conditions. 6
   5.2. Interpretation of the Obtained Chromatograms 6
   5.3. UV-Vis and MS Spectra of the Identified Metabolites 8
6. Biological Evaluation 10
   6.1. Experimental Details of the Photo-cytotoxicity Test 10
   6.2. Results of the (Photo)-cytotoxicity Experiment 10
   6.3. The Selectivity Index 12
   6.4. Interpretation of the Observed Induced Cell Death 12
   6.5. Micropictures of Treated Lung Cancer Cells 13
7. Literature 16

1. General Experimental Procedures.

All used solvents for the preparation of the extracts were purchased from VWR International (Vienna, Austria). Solvents used for HPLC analysis were obtained from Merck (Darmstadt, Germany). Ultrapure water for the HPLC analysis was obtained employing a Sartorius Arium 611 UV water purification system (18MΩ/cm, Sartorius AG, Göttingen, Germany). Solvents used for NMR spectroscopy were purchased from Euriso-top SAS (Saint-Aubin Cedex, France). 9,10-Dimethylanthracene (DMA) was purchased from TCI (TCI Deutschland GmbH, Eschborn, Germany). Berberine was obtained from the stock of standards of the Institute of Pharmacology and Pharmacognosy, University Innsbruck. The *Berberis ilicifolia* root extract was prepared starting with the milled and dried plant material (5 g). The material was sequentially extracted with ethyl acetate (3 x 30 mL) and methanol (3 x 30 mL) in the ultrasonic bath (20 min). The collected extracts were concentrated under vacuum. Berberine was detected in the methanol extract and thus the methanol extract was used.

2. LED Irradiation Setup

0.0	0.2	0.2	0.2	0.3	0.3	0.6	0.0	0.0	0.0	0.0	0.0
0.0	1.1	1.0	1.0	1.0	1.0	1.0	1.0	1.1	1.0	1.1	0.0
0.0	1.1	1.1	1.1	1.1	1.1	1.0	1.0	0.9	1.1	1.0	0.0
0.0	1.0	1.0	1.0	1.1	1.1	1.1	1.0	1.2	1.2	1.1	0.0
0.0	1.0	1.0	1.1	1.1	1.1	1.1	1.1	1.1	1.1	1.0	0.0
0.0	1.0	1.1	1.0	1.0	1.0	1.0	1.1	1.0	1.1	1.0	0.0
0.0	1.0	0.9	1.2	1.3	1.0	1.0	1.0	1.0	1.0	1.0	0.0
0.0	0.1	0.2	0.2	0.3	0.3	0.6	0.0	0.0	0.0	0.0	0.0

Figure S 1 Heat-map showing the equal irradiation (Average = 1.0, STDEV 6.7%) of the inner 60 wells of a 96 well microtiter plate (200 µL). Ferrioxalate was employed as chemical actinometer and an irradiation time of 3 min was chosen. The surrounding outer wells were left empty.

3. Extraction

In Table 1 an overview of the obtained yields is given.

Table 1. Overview of the aceton (AC), petrol ether (PE), and methanol (MeOH) extracts tested.

		Bio-material	PE extract		AC extract		MeOH-Extract	
			yield (rel. yield)	color	yield (rel. yield)	color	yield (rel. yield)	color
Basidiomycetes	*Cortinarius croceus*	1.45 g	8.84 mg (0.006%)	yellowish	23.25 mg (0.020%)	amber	425.32 mg (0.293%)	brown
	Inonotus obliquus	7.00 g	80.15 mg (0.011%)	amber	69.40 mg (0.009%)	yellow	334.6 mg (0.048%)	yellow
	Tapinella atrotomentosa	10.53 g	275.20 mg (0.03%)	cream	829.10 mg (0.080%)	red-brown	2251.0 mg (0.21%)	brown
			Acetone (+1% HCl) Extract					
Ascomycetes	*Beauveria brongniartii*	Complete petri dish (Ø 90 mm) culture	47.77 mg		red-brown			
	Metarhizium brunneum		145.99 mg		orange-green			
	Roesleria subterranea		16.00 mg		green			

4. DMA-Assay

The established DMA-assay is based on the photosensitized oxidation n of $^1O_2$ to 9,10-dimethyl-anthracene disrupting the dislocated π-system (Figure S2) and thereby the typical absorbance of anthracenes.

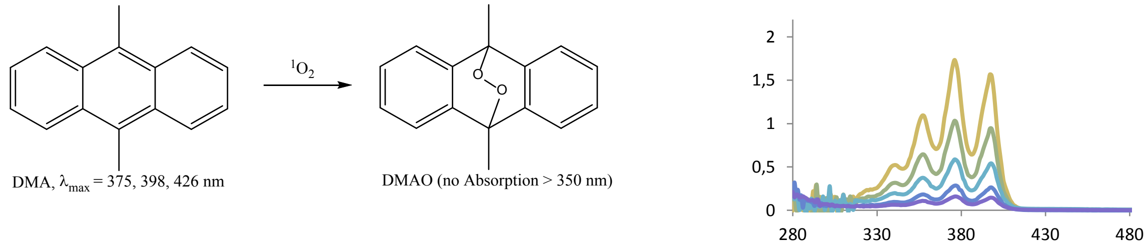

Figure S 2: Left) Chemical reaction of the quenching process. Right) A typical evolution of the electronic absorption spectrum of DMA in ethanol the presence of $^1O_2$ (yellow start, violet end).

4.1. Calculation of the Relative Singlet Oxygen Yield

The relative singlet oxygen yield was calculated as follows in equation (1).

$$^{1}O_2[\%] = \frac{\Delta OD\ (DMA{,}Extract) - \Delta OD\ (DMA{,}Solvent\ CTR)}{\Delta OD\ (DMA{,}Berb) - \Delta OD\ (DMA{,}Solvent\ CTR)} \cdot \frac{1-10^{-OD_{Berb,468\ nm}}}{1-10^{-OD_{Extract,468\ nm}}} \cdot 100 \qquad (1)$$

This equation is derived from the “relative quantum yield measurement method”.[1] Here the quantum yield of an unknown PS is calculated in comparison to a known one. Instead of quantum yields as outcome, we chose “relative singlet oxygen production” ($^{1}O_2$ [%]) as output of our assay to obtain an intuitive measure for the putative phototoxic extracts. In detail, the reduction of the DMA-absorbance by illuminated berberine (EtOH) was normalized to “100% singlet oxygen”. We utilized the absorbance of DMA,[2] instead of the commonly used fluorescence,[3] to avoid false-negative results. The latter would be obtained, if a metabolite of the extract-matrix absorbs the emitted photons of DMA. As the fluorescence emission of DMA lies between at 400 and 450 nm, each yellow extract would lead potentially to a false-negative result. Furthermore, as visualized in equation (1), the difference of the DMA absorbance (i.e. $\Delta OD\ (DMA)$) was corrected by the difference in absorbance of the pure extract at λ = 377 nm (i.e. $OD_{Extract,\ 377\ nm}$). This accounts for potential changes in the OD due to photochemical processes happening in the extract. The second term in equation (1), i.e. $\frac{1-10^{-OD_{Berb,468\ nm}}}{1-10^{-OD_{Extract,468\ nm}}}$, corrects the generation of $^{1}O_2$ by the intensity of absorbed light. This is needed to account for the difference in the concentration of the PSs to compare. With this correction, solely the fraction of molecules being excited is related. The intensity of the absorbed light is derived from Beer-Lambert Law and is depicted in equation (S2), where I_0 equals the incident light.

$$I_{468\ nm\ (Extract)} = I_o \cdot (1 - 10^{-OD_{468\ nm}}) \qquad (S2)$$

As correction, the intensity ratio (i.e. $\frac{I_{468nm\ (Berb)}}{I_{468nm\ (Extract)}}$) was used instead of the commonly used optical density ratio $\left(\frac{OD_{468nm\ (Berb)}}{OD_{468nm\ (Extract)}}\right)$.[4] The latter is an approximated expression of the former[1] and is only valid for OD < 0.2. This assumption is for the investigated extracts in the short-distanced cavity of a 96 well plate generally not valid and therefore negated. Important to note, if another emission source is intended to be used, equation (S2) needs to be adjusted to the emission maximum of the other light source and therewith the measurement. Each measurement was done at least as triplicate of technical duplicates.

5. HPLC-DAD Chromatograms

5.1. Analytical Conditions.

HPLC-DAD-MS experiments were performed on a 1260 Infinity II LC system (Agilent Technology, Waldbronn, Germany) equipped with degasser, binary pump, autosampler, and column heater. The system was fitted with a MS Detector from Agilent. Best separation for the Basidiomycetes was achieved using a Synergi 4µ MAX-RP 80Å (150 mm × 4.6 mm, Phenomenex, Torrance, CA, USA) and utilizing a mobile phase consisting of water (A) and acetonitrile (ACN) with 0.1% formic acid (FA) (B). The linear gradient started with a ratio of 90:10 (A to B), reached after 30 min a ratio of 50:50, after additional 4 min 10:90 and ended with this ratio after 50 min. The column was re-equilibrated for 20 min. Column temperature, flow rate, and sample volume were set to 40 °C, 0.5 mL/min, and 10 µL, respectively. The MS parameters were: split 1:1; API-ES, negative/positive-ion mode; spray voltage, 4.5 kV; dry temperature, 320 °C; drying gas flow rate, 12.00 L/min; nebulizer gas, 25 psi; mode, scan range: m/z 200 – 1200. The ascomycete extracts were investigated with the same MS parameters. However, a different stationary phase (i.e. Luna Phenyl Hexyl column (150 mm x 3.0 mm, 3 µm)) and mobile phase were used. The solvent mixture (H_2O : ACN + 0.1 % FA) started with a ratio of 90:10, reached after 30 min 50:50, after additional 15 min 90:10 and in the last ten minutes 98:2.

5.2. Interpretation of the Obtained Chromatograms

Basidiomycetes. The main peak of *T. atrotomentosa* (Figure S3, t_r = 23 .0 min) correlated with a mass over charge value (m/z) of 323.1 (Figure S5) in the negative mode of the ESI-MS experiment and two absorption maxima at 266 and 371 nm, respectively (Figure S5). Hence, the main pigment was identified as atromentin (Figure S3),[5, 6] which was already described by Kögl et al. in 1924.[7]

The HPLC-chromatogram of *C. croceus* was more complex than the one of *T. atromentosa* (Figure S3). The major peak in the acetone fraction with a retention time of 43.64 min showed a m/z value of 565 for $[M-H]^-$ (Figure S6). This, in combination with the taxonomic description of *Cortinariu*s subgenus *Dermocybe* [8] and the electron-absorption properties (i.e. λ_{max} = 284*s*, 310*sh*, 440*br* nm, see Figure S6) led to the identification of 7,7'-biphyscion (Figure S6) as main-pigment of these fruiting bodies. Interestingly, this dianthraquinone is not genuinely present in this fungus. Rather it is an oxidation product from a flavomannin-precursor.[9]

The identification of the main pigment of the third basidiomycete, *I. obliquus*, was not possible with the current experimental setup. For this organism, the HPLC-chromatogram showed one main peak in the acetone fraction with a retention time of t_R = 25.2 min, which was surrounded by several minor peaks (see Figure S3). The major peak was characterized with m/z = 379.2 $[M-H]^-$ and the following electron-absorption properties, i.e. λ_{max} = 412 nm. None of the pigments known for *I. obliquus*, i.e. phelligridins or innonoblins,[10, 11] correlated with these characteristics.

Ascomycetes. The chromatograms detected at 468 nm are depicted for all fungi in Figure S4. For *B. brongniartii* one peak was detected in the polar region at t_R = 17.2 min. This peak was assigned to the red pigment of *B. brongniartii* (i.e. oosporein) via comparing its retention time, UV-Vis spectrum (λ_{max} = 288, 501 nm) and MS-spectrum (m/z (-) = 305.1) with the reference compound oosporein (Figure S7).

The orange-green extract of *M. brunneum* was characterized by a small peak eluting at t_R = 11.8 min. The UV-Vis spectrum showed a λ_{max} = 410 nm in the visual range (Figure S8 left). The ionization of the metabolite was not good enough to gain a reliable m/z value for this metabolite, most likely it is an unknown one.

The *R. subterranea* extract (see Figure S7) is due to its complex pigment pattern the most interesting one. The three main pigments absorbing at 468 nm elute at t_R = 29.4 min, 34.8 min, and 47.9 min. The UV-Spectra are characterized by a broad absorbance band between 330 and 500 nm (see Figure S8 right), which is characteristic for phenalenone like structures and fits to the vast number of poly-hydroxylated phenalenones and phenalenone-like structure described for *Roesleria* and related fungi [12-14].

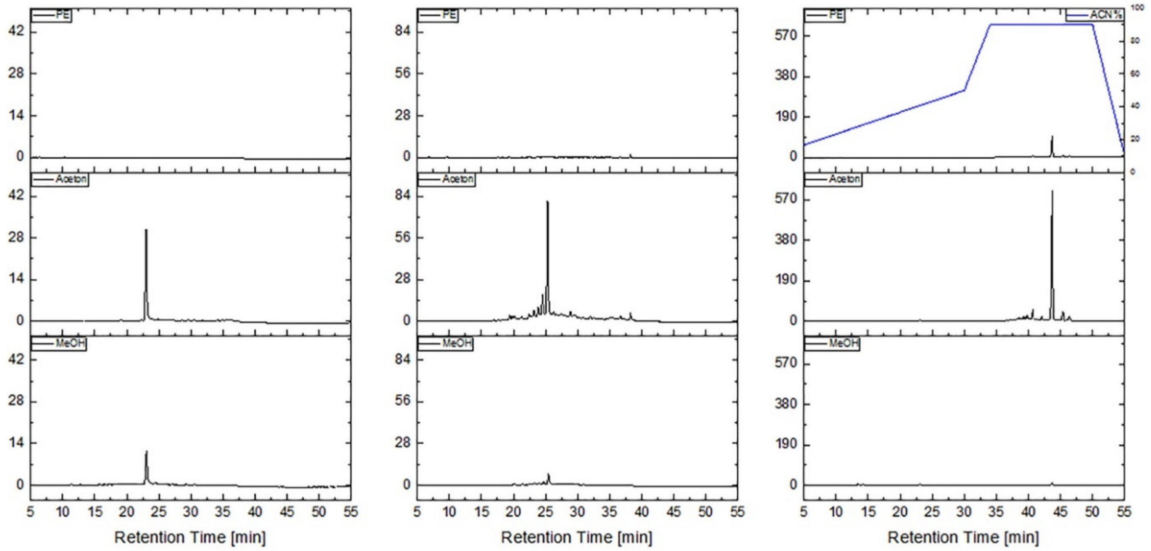

Figure S 3. HPLC-DAD chromatograms recorded at 468 nm for the extracts of Tapinella atrotomentosa (left), Inonotus obliquus (middle), and Cortinarius croceus (right). To represent the amount of pigments per extract the y-axis were aligned for each fungi. As solid phase, Synergi Max RP was used. The mobile phase consisted out of water and acetonitrile supplemented with 0.1 % formic acid. A gradient (blue line) as indicated in the upper right chromatogram was employed.

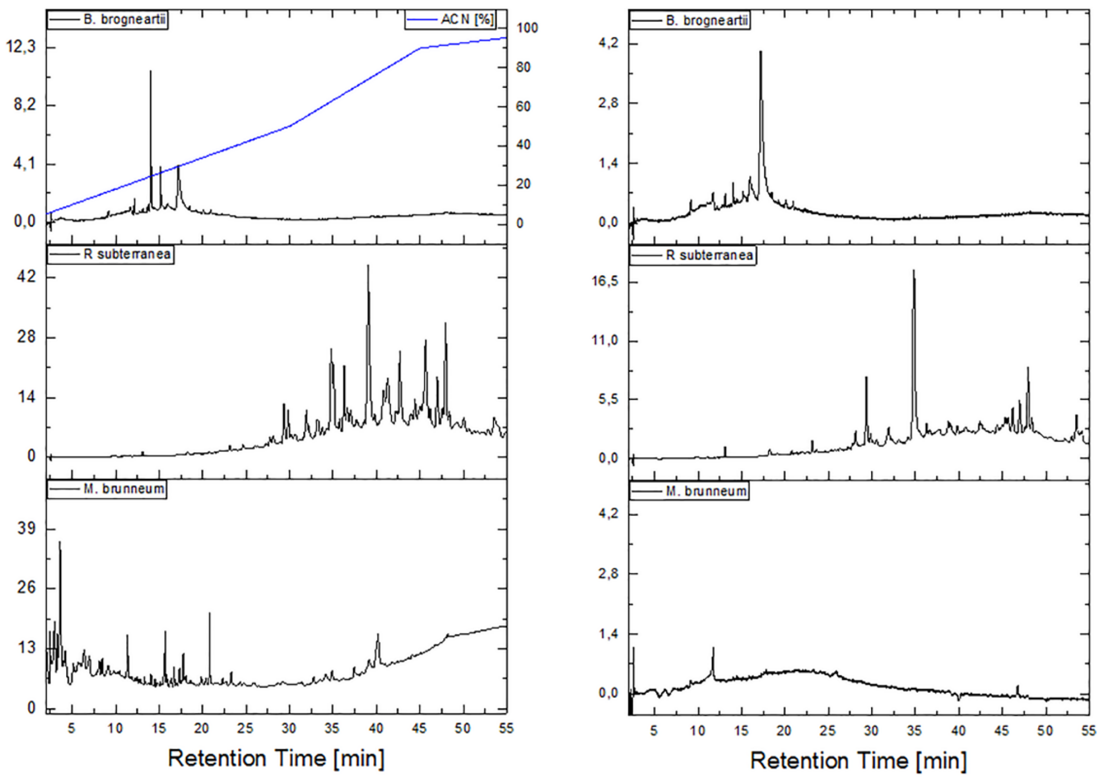

Figure S4 HPLC-DAD chromatograms recorded at 428 nm (left) and 468 nm (right) for the extracts of the ascomycetes, as indicated in the legend. As solid phase a Luna Phenylhexyl column was used. The mobile phase (0.5 mL/min) consisted out of water and acetonitrile supplemented with 0.1 % formic acid. A gradient as indicated (blue line) in the upper left chromatogram was employed.

5.3. UV-Vis and MS Spectra of the Identified Metabolites

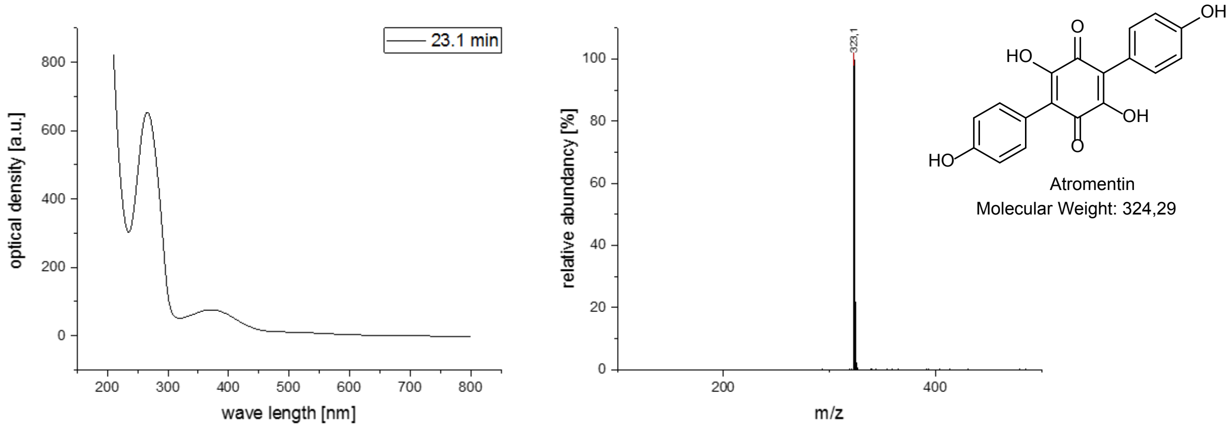

Figure S 5. Left) Electron absorption spectra of atromentin, the main metabolite in the extract of T. atromentosus eluting at t_r = 23.1 min. Right) The respective mass spectrum of atromentin.

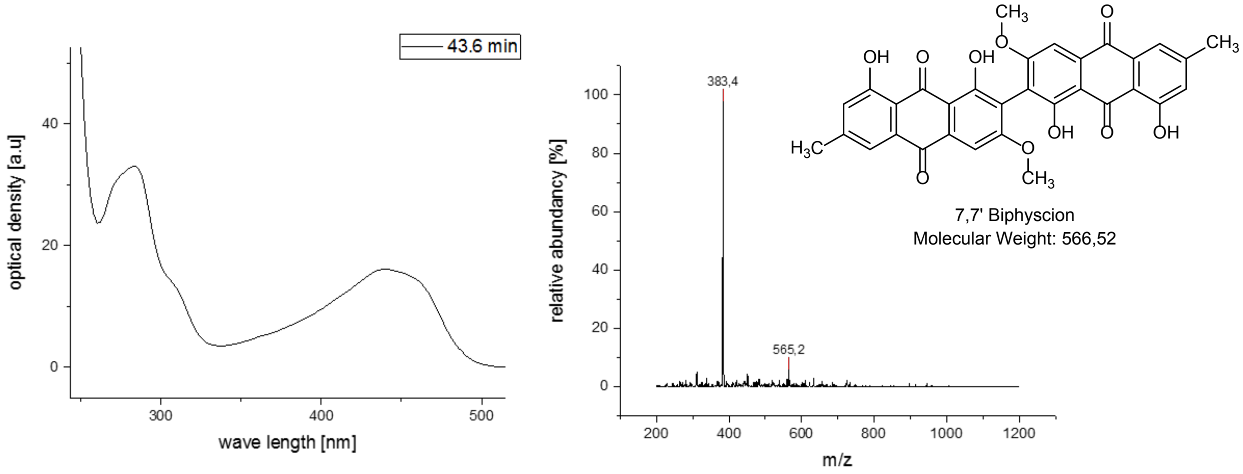

Figure S 6. Left) Electron absorption spectra of 7,7' biphyscion, the main metabolite in the extract of C. croceus eluting at t_r = 43.6 min. Right) The respective mass spectrum of 7,7'-biphyscion.

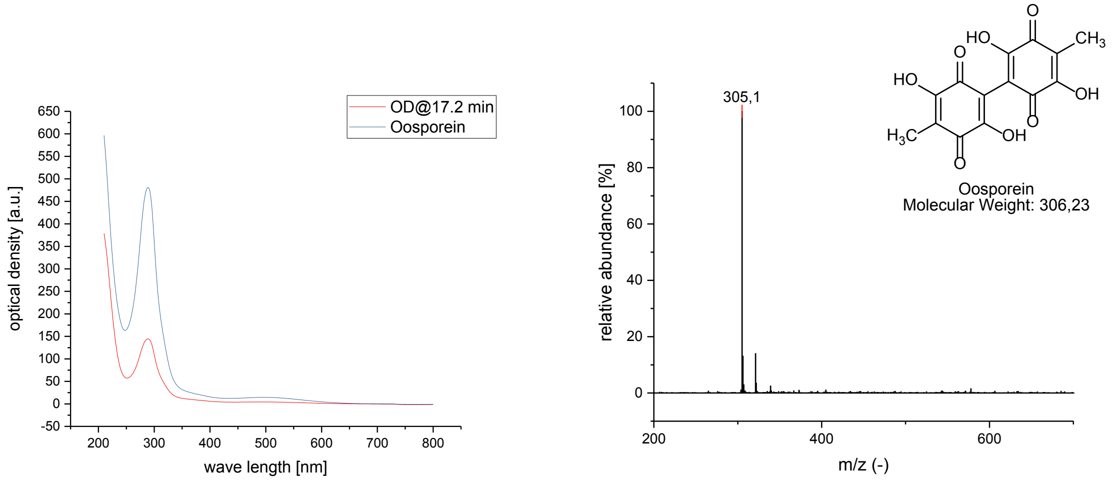

Figure S 7 Left) Electron absorption spectra oosporein (blue) and the main metabolite in the extract of B. brongniartii eluting at t_r = 17.2 min. Right) the respective mass spectrum of this metabolite.

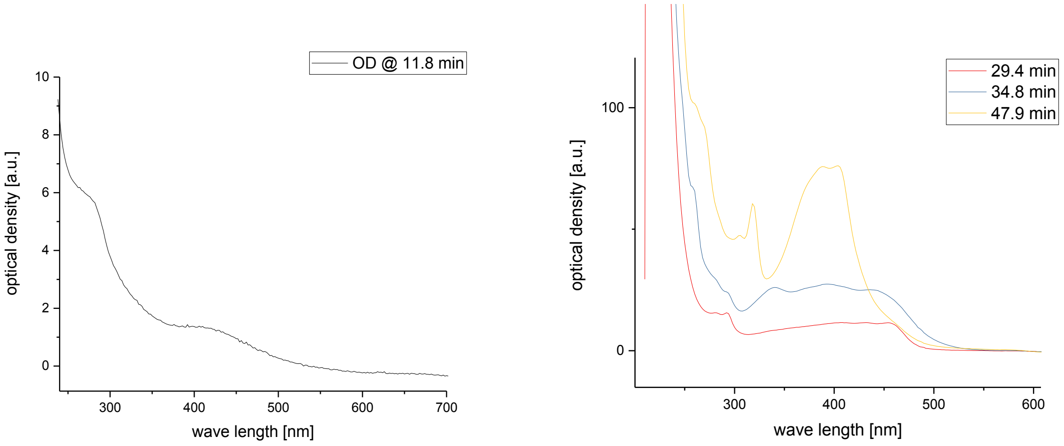

Figure S 8. Left) UV-Vis spectra of the metabolites from M. brunneum eluting at t_R = 11.8 min and absorbing at 468 nm. Right) UV-Vis spectra of the main-metabolites from R. subterranea absorbing at 468 nm.

6. Biological Evaluation

6.1. Experimental Details of the Photo-cytotoxicity Test

Briefly, cells (2000/well) were seeded in Opti-MEM® (2.5% FCS, P/S) and after 24 h treated with each extract (50, 25, and 5 µg/mL, stock solution 10 mg/mL in DMSO, max DMSO 0.65%). After additional 24 h, the medium was aspirated and replaced by fresh Opti-MEM®. Thereafter, the plate was irradiated for the indicated amount of time (i.e. 0, 2.5, 5, or 7.5 min; 0, 3.1, 6.2, or 9.3 J/cm² respectively). The cells were fixed with chilled trichloracetic acid (100 µL) after 72 h in total. After washing (4 times 200 µL, water) the cells were stained with sulforhodamine B (SRB, 0.4 % in acetic acid (1 %)) for 30 min. Then, the plates were washed again (4 times 200 µL, acetic acid 1 %) and tried under air. The dried dye was dissolved with a TRIS solution (10 mM in water, 100 µL) and the absorbance measured at 540 nm with a plate reader (Tecan, Spark, M10). The EC_{50} of berberine was calculated with Prism 5.0 employing the relative Hill-Slope equation and is given with its confidence interval (95 %).

6.2. Results of the (Photo)-cytotoxicity Experiment

The detailed results of the (photo)-cytotoxicity experiment are given in Table S2.

Table S2. List of EC_{50} in µg/mL as obtained for the fungal extracts. The values are given with their upper and lower confidence interval (95 %). PE, AC, MeOH indicate the original solvent used to prepare the investigated extract.

EC50 [µg/mL]		A549								HeLa			
		Dark		3.1 J/cm²		6.2 J/cm²		9.3 J/cm²		Dark		3.1 J/cm²	
T. atrotomentosa	PE	23	22<br>11	39	8<br>7	40	9<br>7	42	6<br>5	34	3<br>3	36	3<br>3
	AC	> 50		> 50		> 50		> 50		> 50		> 50	
	MeOH	> 50		> 50		> 50		> 50		> 50		> 50	
I. obliquus	PE	6	2<br>2	8	1<br>1	7	1<br>1	7	1<br>1	11	3<br>2	11	3<br>2
	AC	18	15<br>8	28	8<br>6	27	10<br>7	21	13<br>8	13	6<br>3	14	5<br>3
	MeOH	> 50		> 50		> 50		> 50		> 50		>50	
C. croceus	PE	29	9<br>7	9	2<br>2	7	1<br>1	4	1<br>1	29	3<br>2	12	3<br>3
	AC	> 50		7	3<br>2	3	3<br>1	1	1<br>0	> 50		9	5<br>3
	MeOH	> 50		> 50		16	3<br>2	9	1<br>1	> 50		> 50	
R. subterranea	AC (1% HCl)	29	3<br>3	24	2<br>2	16	2<br>2	14	1<br>1	27	2<br>2	21	3<br>3
B. brongniartii	AC (1% HCl)	53	15<br>12	50	5<br>5	42	10<br>8	32	13<br>9	> 50		> 50	
M. brunneum	AC (1% HCl)	> 50		> 50		> 50		> 50		> 50		> 50	
B. ilicifolia	MeOH	> 50		31		22		17		> 50		33	
Berberine		> 50		7	4<br>3	4	2<br>1	2	1<br>1	> 50		14	2<br>2

6.3. The Selectivity Index

The selectivity indices of the extracts were calculated as discussed in the experimental part and are presented in Figure S9

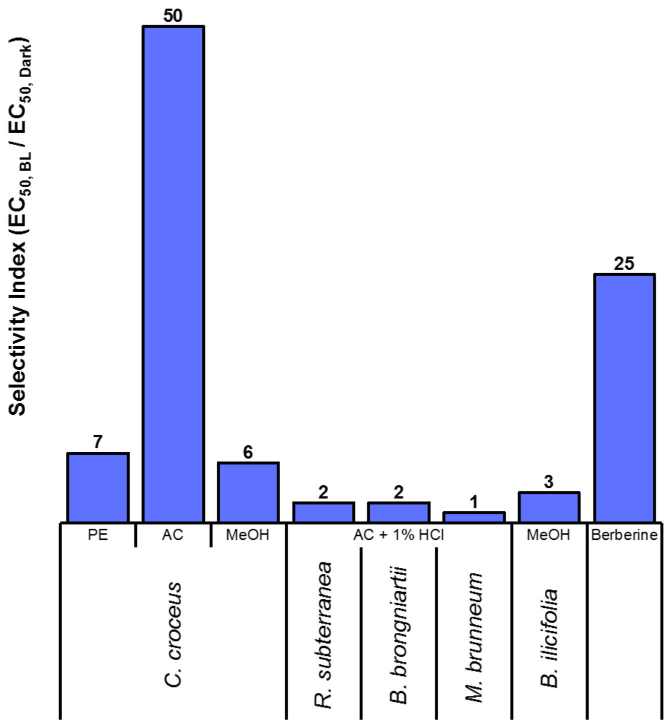

Figure S 9. Approximated selectivity index (SI; A549 cells) of the phototoxic extracts at the highest light dose (i.e. 9.3 J/cm²). A value of 50 µg/mL was estimated for those extracts to which no exact dark cytotoxicity was determined (labeled with >50 µg/mL in Table 2). For these extracts, the calculated SI-values are therefore likely an underestimation of the real SI.

6.4. Interpretation of the Observed Induced Cell Death

A systematic investigation of the morphological changes induced by the extracts and the irradiation is depicted in Figure 3 and Figure S10-S15. Lung cancer cells treated with the AC extract of *C. croceus* (Figure 3A, Figure S14) did significantly shrink and do otherwise show all hallmarks of an apoptotic cells death indicating that the PS accumulated primarily within the organelles. In contrast, cell populations treated with *I. obliquus* PE extract showed dispatched cells, called balloon-cells, and a lot of cell debris (Figure S13). The swelling of the cells is a typical observation for membrane modulating agents, which break down the cellular membrane and therewith yield the loss of the osmotic balance. The PE extract of *T. atrotomentosa* induced as well in the dark as in the light a programmed cell death as observable by the shrunken cells and the absence of debris. In addition, the irradiated cells treated with *R. subterranea* showed apoptosis-typical membrane blebbing, whereas the extract of *B. brongniartii* induced rather an

antiproliferative effect as the number of shrunken cells does not out count the number of still normal cells in the irradiation experiment. *M. brunneum* does not show any distinct conspicuity.

In sum, the phototoxic evaluation of the fungal extract indicated the existence of promising fungal PS with impressive selectivity indices and a putative apoptotic mode-of-action. The latter is clearly medicinally favored, due to its lack of inflammatory processes.

6.5. Micropictures of Treated Lung Cancer Cells

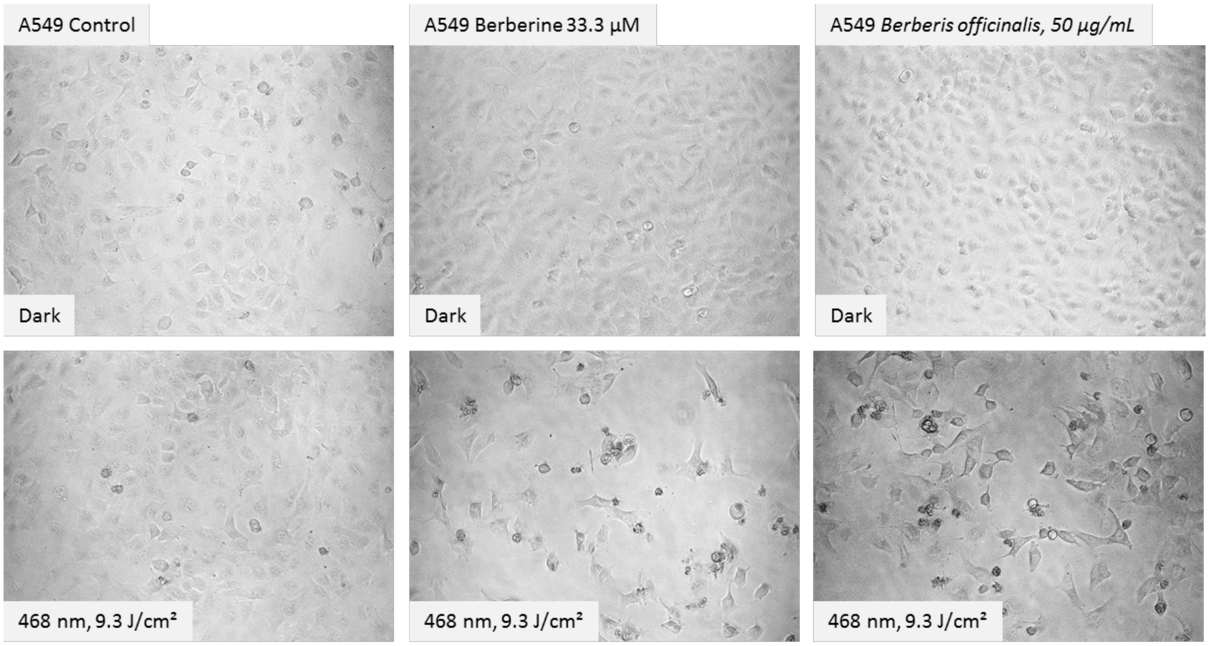

Figure S 10. Cells of the A549 lung cancer cell-line treated with Berberine and a B. ilicifolia extract. Pictures were taken 48 h after irradiation.

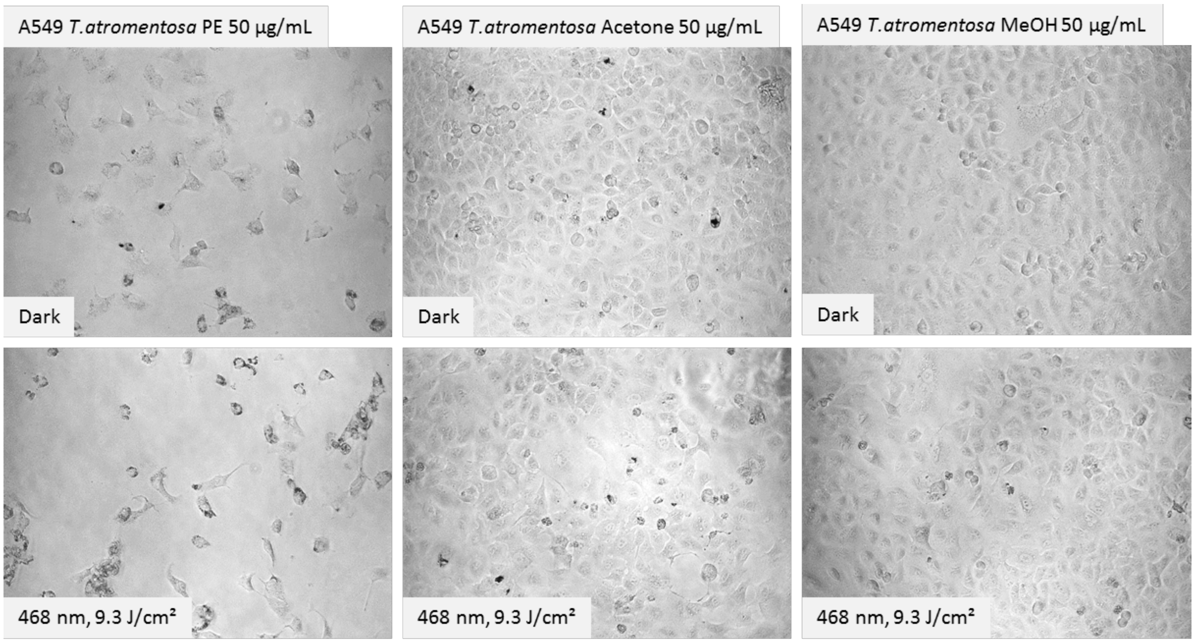

Figure S 11. Cells of the A549 lung cancer cell-line treated with T. atromentosa extracts. Pictures were taken 48 h after irradiation.

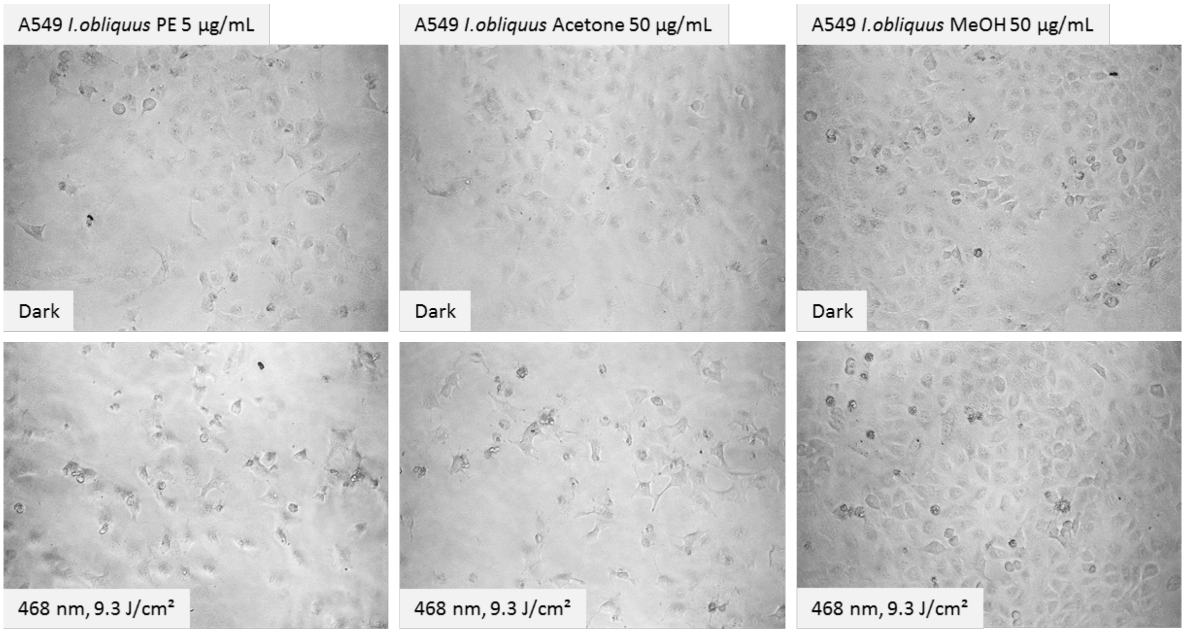

Figure S 12 Cells of the A549 lung cancer cell-line treated with I. obliquus extracts. Pictures were taken 48 h after irradiation.

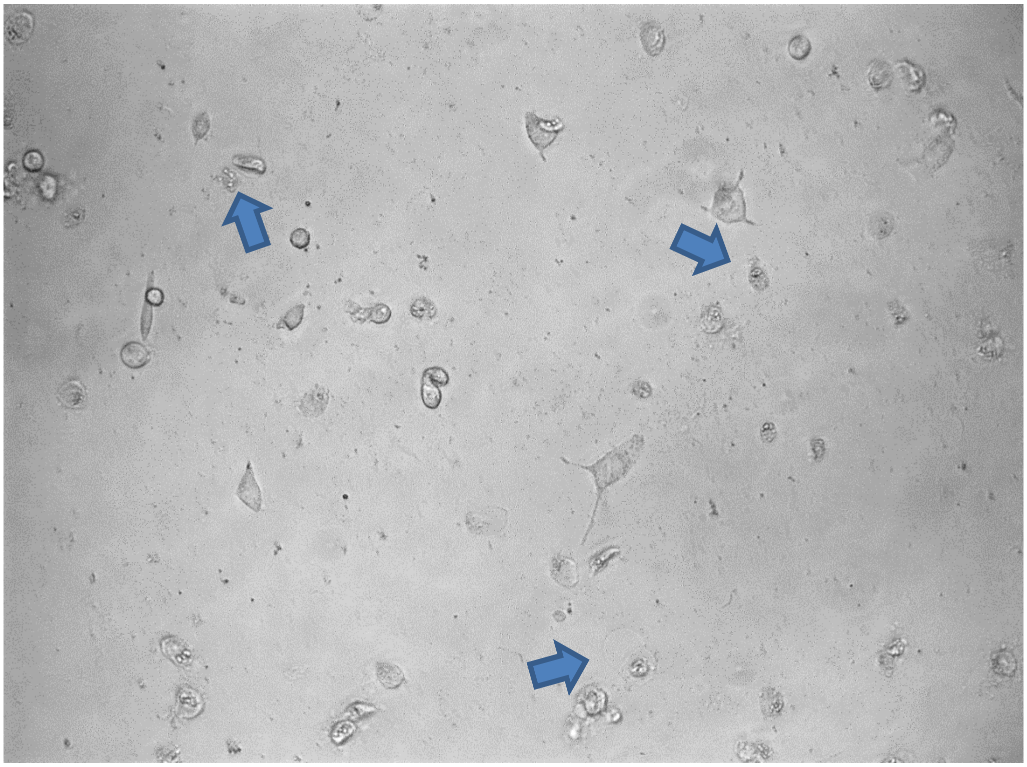

Figure S 13. A549 cells treated with the PE extract of I. obliquus (50 µg/mL) in the dark. Highlighted with arrows are so-called "balloon"-cells indicating the damage of the cell membrane.

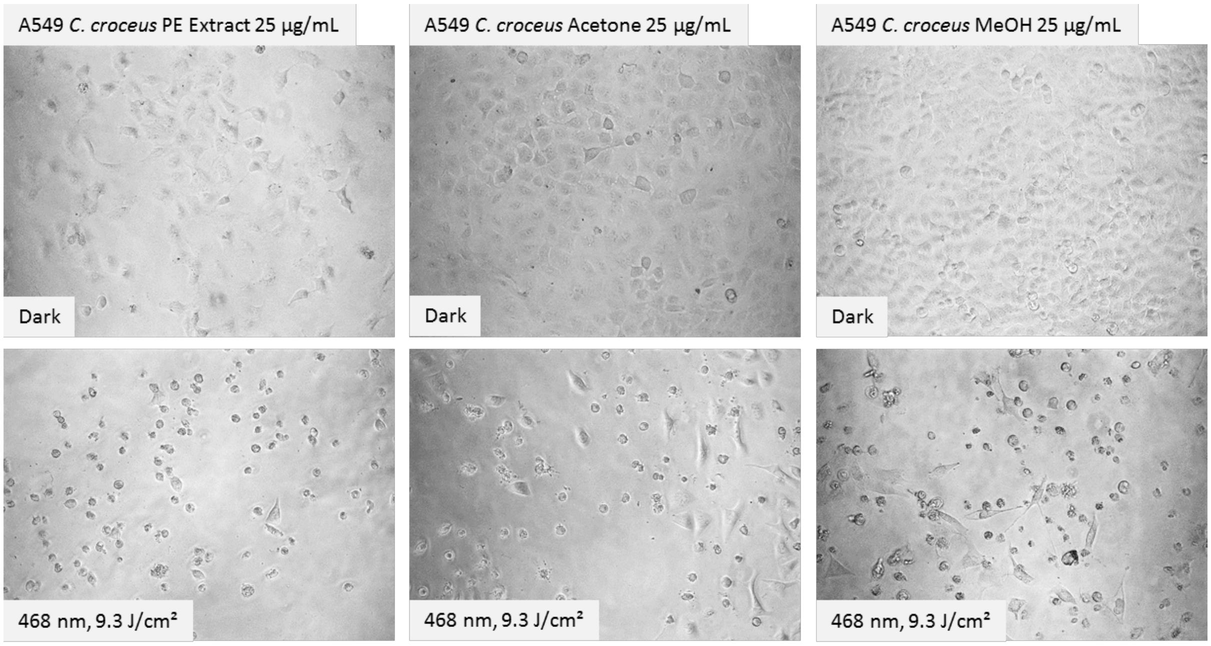

Figure S 14 Cells of the A549 lung cancer cell-line treated with C. croceus extracts. Pictures were taken 48 h after irradiation.

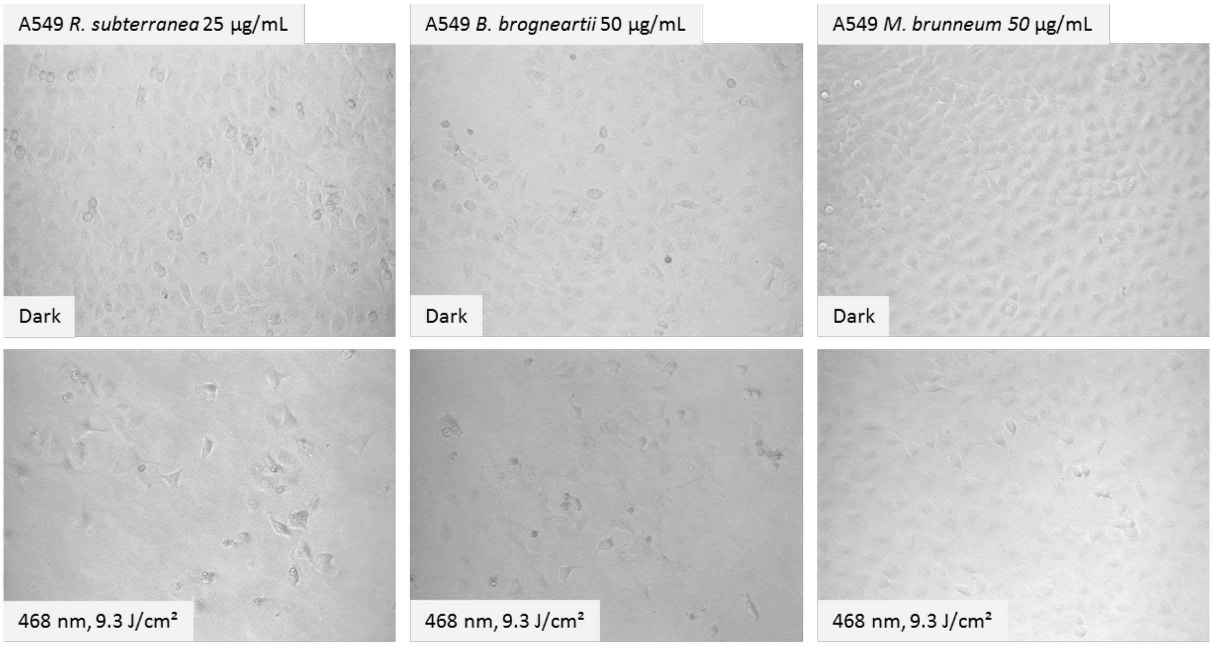

Figure S 15 Cells of the A549 lung cancer cell-line treated with the extracts of the investigated ascomycetes. Pictures were taken 48 h after irradiation.

7. Literature

1. Crosby, G. A.; Demas, J. N., Measurement of photoluminescence quantum yields. Review. *Journal of Physical Chemistry* **1971,** 75, (8), 991-1024.

2. Waiblinger, F.; Keck, J.; Fluegge, A. P.; Kramer, H. E. A.; Leppard, D.; Rytz, G., Ultraviolet absorbers and singlet oxygen. *J. Photochem. Photobiol., A* **1999,** 126, (1-3), 43-49.

3. Gomes, A.; Fernandes, E.; Lima, J. L. F. C., Fluorescence probes used for detection of reactive oxygen species. *Journal of Biochemical and Biophysical Methods* **2005,** 65, (2–3), 45-80.

4. Fery-Forgues, S.; Lavabre, D., Are Fluorescence Quantum Yields So Tricky to Measure? A Demonstration Using Familiar Stationery Products. *Journal of Chemical Education* **1999,** 76, (9), 1260.

5. Hu, L.; Liu, J.-K., p-Terphenyls from the basidiomycete Thelephora aurantiotincta. *Z. Naturforsch., C: J. Biosci.* **2003,** 58, (5/6), 452-454.

6. Schneider, P.; Bouhired, S.; Hoffmeister, D., Characterization of the atromentin biosynthesis genes and enzymes in the homobasidiomycete Tapinella panuoides. *Fungal Genet. Biol.* **2008,** 45, (11), 1487-1496.

7. Kogl, F.; Postowsky, J. J., Fungi dyestuffs. I. Atromentin. *Ann.* **1924,** 440, 19-35.

8. Arnold, N.; Bresinsky, A.; Kemmer, H., Notizen zur Chemotaxonomie der Gattung Dermocybe (Agaricales) und zu ihrem Vorkommen in Bayern. *Zeitschrift für Mykologie* **1987,** 53, (2), 187-194.

9. Steglich, W.; Töpfer-Petersen, E.; Reininger, W.; Gluchoff, K.; Arpin, N., Isolation of flavomannin-6,6′-dimethyl ether and one of its racemates from higher fungi. *Phytochemistry* **1972,** 11, (11), 3299-3304.

10. Lee, I.-K.; Yun, B.-S., Styrylpyrone-class compounds from medicinal fungi Phellinus and Inonotus spp., and their medicinal importance. *Journal Of Antibiotics* **2011,** 64, 349.

11. Lee, I.-K.; Kim, Y.-S.; Jang, Y.-W.; Jung, J.-Y.; Yun, B.-S., New antioxidant polyphenols from the medicinal mushroom Inonotus obliquus. *Bioorganic & Medicinal Chemistry Letters* **2007,** 17, (24), 6678-6681.

12. Elsebai, M. F.; Saleem, M.; Tejesvi, M. V.; Kajula, M.; Mattila, S.; Mehiri, M.; Turpeinen, A.; Pirttila, A. M., Fungal phenalenones: chemistry, biology, biosynthesis and phylogeny. *Natural Product Reports* **2014,** 31, (5), 628-645.

13. Bachmann, O.; Kemper, B.; Musso, H., The Green Pigment from the Fungus Roesleria-Hypogea. *Liebigs Annalen Der Chemie* **1986**, (2), 305-309.

14. Robinson, N.; Wood, K.; Hylands, P. J.; Gibson, T. M.; Weedon, C. J.; Covill, N., Blue pigments of Penicillium herquei. *J. Nat. Prod.* **1992,** 55, (6), 814-17.
